# Putative Effects of an Orally Administered Anti-TLR4 Antibody on Gut Microbiota and Acetaminophen Hepatotoxicity in Mice

**DOI:** 10.1128/spectrum.01863-22

**Published:** 2022-12-13

**Authors:** Hartmut Jaeschke, Anup Ramachandran

**Affiliations:** a Department of Pharmacology, Toxicology and Therapeutics, University of Kansas Medical Center, Kansas City, Kansas, USA; University of Nebraska—Lincoln

**Keywords:** acetaminophen, drug hepatotoxicity, gut microbiome

## LETTER

We read with interest the recent paper by Sun et al. (1) showing that an anti-TLR4 antibody protected against acetaminophen (APAP)-induced liver injury by modulating gut microbiota and gut permeability. At first glance, these results may provide an alternate explanation for the long-term controversy about the role of inflammation in the injury process (2). However, severe concerns emerged. First, the authors administered the antibody orally to fed animals. The authors did not assess or even discuss how the antibody, which is a protein, when administered into the stomach of a fed mouse, can survive digestive enzymes intact to influence the microbiome and gut permeability.

In addition, the authors did not use an appropriate control (isotype-matched IgG) in these experiments. Both of these issues raise serious concerns regarding the mechanistic interpretation of the results and the potential for off-target effects with this intervention.

Second, the authors administered the antibody 2 h after APAP. By then, hepatic glutathione (GSH) levels are depleted by 90% by the reactive metabolite and mitochondrial oxidant stress and c-jun N-terminal kinase (JNK) activation and mitochondrial translocation has occurred. Subsequent peroxynitrite formation and mitochondrial dysfunction cause centrilobular necrosis within 6 to 12 h after an APAP overdose (3). The critical role of these events in APAP-induced cell death and liver injury in mice and in humans is highlighted by the efficacy of clinically used drugs, such as *N*-acetylcysteine and fomepizole, which promote hepatic GSH synthesis to scavenge the reactive metabolite and peroxynitrite and inhibit Cyp2E1 and JNK, respectively (4). The therapeutic window for these highly effective, direct-acting drugs in the mouse model is less than 6 h after treatment with 600 mg/kg of body weight APAP (4, 5). The authors now suggest that APAP changes gut microbiota composition and enhances gut permeability, with microbial metabolites contributing to APAP-induced liver injury (1). Importantly, an anti-TLR4 antibody administered orally 2 h after APAP escapes digestion and prevents these events in the intestine and colon (1). For one, the authors showed changes in gut microbiota and gut permeability at 24 h after APAP, i.e., long after the liver injury has occurred. This raises fundamental concerns regarding their contribution to liver injury, given the known timeline of events of APAP hepatotoxicity (6).

Third, the authors attempted to mimic the effects of the antibody by using fecal microbiota transplantation for 3 days or short-chain fatty acids treatment for 7 days (1). Thus, the question arises why these treatments had to be done for several days compared to a single posttreatment with the antibody? This again, raises concerns regarding the antibody treatment. Furthermore, the authors did not assess effects of these pretreatments on drug metabolism and adaptive genes that could be the actual cause of protection. Thus, the protective mechanisms of the pretreatment interventions remain unclear and any relevance for the antibody effects is questionable.

In summary, the results presented by Sun et al. (1) raise serious concerns regarding the data and their interpretation, as no evidence was provided as to how an antibody can survive the digestive tract intact and realistically influence the pathophysiology of APAP hepatotoxicity within a relevant time frame.
